# Direct medical cost associated with diabetic retinopathy severity in type 2 diabetes in Singapore

**DOI:** 10.1371/journal.pone.0180949

**Published:** 2017-07-10

**Authors:** Xiao Zhang, Serena Low, Neelam Kumari, Jiexun Wang, Keven Ang, Darren Yeo, Chee Chew Yip, Subramaniam Tavintharan, Chee Fang Sum, Su Chi Lim

**Affiliations:** 1 Clinical Research Unit, Khoo Teck Puat Hospital, Singapore, Republic of Singapore; 2 Department of ophthalmology and Visual Sciences, Khoo Teck Puat Hospital, Singapore, Republic of Singapore; 3 Department of Medicine, Khoo Teck Puat Hospital, Singapore, Republic of Singapore; 4 Diabetes Centre, Khoo Teck Puat Hospital, Singapore, Republic of Singapore; Soochow University Medical College, CHINA

## Abstract

Diabetic retinopathy (DR) is a leading cause of vision-loss globally among type 2 diabetes (T2DM) patients. Information on the economic burden of DR in Singapore is limited. We aim to identify the total annual direct medical costs of DR at different stages, and to examine factors influencing the costs. Four hundreds and seventy T2DM patients who attended the Diabetes Centre in a secondary hospital in Singapore in 2011–2014 were included. Digital color fundus photographs were assessed for DR in a masked fashion. Retinopathy severity was further categorized into non-proliferative DR (NPDR), including mild, moderate and severe NPDR, and proliferative DR (PDR). Medical costs were assessed using hospital administrative data. DR was diagnosed in 172 (39.5%) patients, including 51 mild, 62 moderate and 18 severe NPDR, and 41 PDR. The median cost in DR [2012.0 (1111.2–4192.3)] was significantly higher than that in non-DR patients [1158.1 (724.1–1838.9)] (p<0.001). The corresponding costs for mild, moderate, severe NPDR and PDR were [1167.1 (895.4–2012.0)], [2212.0 (1215.5–3825.5)], [2717.5 (1444.0–6310.7)], and [3594.8.1 (1978.4–8427.7)], respectively. After adjustment, the corresponding cost ratios for mild, moderate, severe NPDR, and PDR relative to non-DR were 1.1 (p = 0.827), 1.8 (p = 0.003), 2.0 (p = 0.031) and 2.3 (p<0.001), respectively. The other factors affecting the total cost include smoking (ratio = 1.7, p = 0.019), neuropathy (ratio = 1.9, p = 0.001) and chronic kidney disease (CKD) (ratio = 1.4, p = 0.019). The presence and severity of DR was associated with increased direct medical costs in T2DM. Our results suggest that preventing progression of DR may reduce the economic burden of DR.

## Introduction

Type 2 diabetes mellitus (T2DM) is a rapidly evolving global health issue and Asia is the epi-center of this worldwide epidemic [1,2]. In Singapore, T2DM prevalence has been predicted to double from 7.3% in 1990 to 15% in 2050 [3]. Recently, Png et al. estimated that total of direct medical cost and indirect productivity-related cost for working-age T2DM patients in Singapore is US$787 million in 2010 and expected to rise to US$1867 million in 2050. The total cost per patient is estimated to be US$5646 in 2010 and US$7791 in 2050 [4]. In 2010, the total cost for diabetes accounts for almost 10% of health care spending and has imposed a significant economic burden on the national health care system [4].

The occurrence of major diabetes-related complications further aggravated the economic impact of diabetes [5–7]. Diabetic retinopathy (DR) is a common microvascular complications of T2DM and is a leading cause of irreversible blindness among adults of working age [8]. The overall prevalence of DR is lower in Asians (12.1–23.0%) compared with Western population (28.5–43.5%) [9]. However, Singapore is a notable exception to this trend. DR prevalence in Singapore is higher (33.9%) than other Asian countries but comparable to Western populations [10].

Despite the high DR prevalence, the information on its financial burden in Singapore is still lacking. Currently, most cost-of-illness studies of DR have been carried out in Western countries. Four studies in Europe and USA have reported association of higher medical cost with the presence or severity of DR [11–14]. A study in Sweden further demonstrated that the cost difference is attributable to diagnosing or treating DR [15]. A longitudinal study in Taiwan reported association of direct medical cost with severity of DR [16]. In addition, the progression from early to advanced stage of DR experienced the greatest increase in cost compared with patients without progression [16]. In a recent study among T2DM patients in Singapore, the annual direct medical cost was found to be US$1575.6 (SGD2034.6) [17]. T2DM complications, such as nephrology and cardiovascular diseases, are independent factors influencing the total costs. Although the cost ratio for DR relative to non-DR patients were 1.12, the association failed to reach statistical significance, possibly because of relative small sample size for DR patients [17].

The aim of study is to estimate the direct medical costs based on the actual expenses incurred, which was retrieved from the hospital administrative data. We also aim to evaluate the association of the cost with the presence and severity of DR.

## Materials and methods

### Study population and design

The study was approved by the National Healthcare Group Domain Specific Review Board (NHG-DSRB) and conducted in accordance with the Declaration of Helsinki. This study adopted a prevalence-based epidemiological approach using a bottom-up methodology to estimate different cost components, an approach to yield precise estimates by ascertaining the current rather than projected economic burden [18,19]. Written informed consent was obtained from all subjects prior to enrollment in the study.

We have included 482 subjects with digital color fundus photographs available for assessed for the presence and severity of DR in a masked fashion from the Singapore Study of Macro-angiopathy and Micro-vascular Reactivity in Type 2 Diabetes (SMART2D), a cross-sectional study of 2,057 adults aged 21–90 years with T2DM that was conducted between August 2011 and February 2014. The methodology of SMART2D has been previously described in detail elsewhere [20]. Generally, these 482 selected subjects have similar profile compared to unselected subjects in SMART2D, except disease burden (S1 Table). Of the 482 subjects, 47 subjects were not included due to the following reasons: T1DM (n = 3); missing clinical information (n = 1); non gradable photos (n = 14); and missing medical cost information (n = 29). These 47 patients have generally similar profile as the 435 subjects, who were finally included in the analysis (S2 Table).

### Assessment of DR

Non-mydriatic digital images of the retina for both eyes were taken in all study subjects using a retinal camera (TRC-NW 200, Topcon Co Japan). Digital color fundus photographs were assessed for the presence of DR by a fellowship trained retina specialist in a masked fashion to minimize any possible bias. The photographs were not graded and labeled as ungradable if more that 50% of the retinal photographs were not clearly visible.

DR was considered present if any characteristic lesions as defined by Early Treatment Diabetic Retinopathy Study (ETDRS) were present. The minimum criterion for diagnoses of DR was presence of at least one definite micro aneurysm and/or retinal hemorrhage. DR severity was further categorized into mild, moderate and severe non-proliferative DR (NPDR) and proliferative DR (PDR) [21]. DR was classified as NPDR based on the presence of one or more of the following features: micro aneurysms, hemorrhages, hard or soft exudates, venous beading, and intraretinal microvascular abnormalities. DR was classified as PDR if there was neovascularization, pre-retinal hemorrhages, vitreous hemorrhage, or pan retinal laser photocoagulation scars. The severity of DR in the worse affected eye was used for retinopathy grading.

### Cost data collection

Annual direct medical costs were assessed using hospital administrative data from 2011 to 2014. The costs were classified by the type of service, including inpatient hospitalization, accident and emergency (A&E) and ambulatory outpatient care (physician visits, allied health visits, laboratory tests and medications) [17]. Allied health visits include foot screening, eye screening, dietary services and health education. The cost for drugs other than anti-diabetic medications was not included. Any A&E visits that resulted in hospitalization were included as inpatient care costs. Direct non-medical costs (i.e., transportation expense) and indirect costs (i.e., early retirement, lost productivity) were not included.

Direct medical costs were measured from the hospital perspective by using the total before-subsidy charges, which is the total medical bill before any deduction for government subsidies or insurance claims. All costs were expressed in year 2014 Singapore dollars (SGD). Consumer price index was used to estimate values older than 2014.

### Clinical and biochemical measurement

Body mass index (BMI) was calculated as body weight (kg)/height (m)^2^. Hemoglobin A1c (HbA1c) was measured based on monoclonal antibody agglutination reaction using a point-of-care immunoassay analyzer (DCA Vantage Analyzer; Siemens, Erlangen, Germany) certified by National Glycohemoglobin Standardization Program. Urinary albumin-to-creatinine ratio (ACR) was determined by urinary creatinine measured by enzymatic method on Roche/Hitachi cobas c system (Roche Diagnostic GmbH, Mannheim, Germany) and albumin measured by a solid-phase competitive chemiluminescent enzymatic immunoassay with a lower detection limit of 2.5 μg/ml (Immulite; DPC, Gwynedd, UK). Estimated GFR (eGFR) was calculated based on a widely used Modified Diet in Renal Disease equation in patients with diabetes [22]. Chronic kidney disease (CKD) was defined by the presence of eGFR <60 ml/min/1.73m^2^ or kidney damage as indicated by ACR≥30mg/g [23]. Assessment for neuropathy was performed by a neurothesiometer (Horwell Scientific, Wilford, Nottingham, UK) for vibration and with a 10-g monofilament for light touch as described previously [24]. Neuropathy was present if an abnormal finding in monofilament (inability to detect at least 8 of 10 points on either foot) or neurothesiometer testing of ≥ 25 V was detected [24].

### Statistical analysis

Standard descriptive statistics were used to describe the characteristics of T2DM patients with and without DR. Normally distributed continuous data were expressed as means and standard deviations (SDs). The skewed medical costs data were expressed as median and inter-quartile range (IQR). Differences between DR and no DR patients were compared by t-test, Wilcoxon rank-sum test, or *χ*^2^ test where appropriate. For the severity analysis among subgroups in DR patients, differences in medical cost were compared with the Kruskal-Wallis test.

The medical cost in our study is highly skewed. Traditional models for skewed cost data are ordinary least squares regressions with a simple logarithmic (log) transformation in order to normalize cost data [25–27]. However, this method usually induces symmetry but not normality [28]. Prior studies have found that back transformation is a complicated model-dependent re-transformation process rather than simple exponentiation [29,30]. The interpretation of regression coefficients for log-transformed variables may not be intuitive, especial for variables with heteroscedastic errors (i.e., log-scale variances of errors between groups are not equal) [28]. Generalized linear models (GLM) addresses linearity in cost variable on the specified scale and accommodates skewness through variance weighting. Unlike log transformation-based methods, estimates of parameters using GLM were directly obtained from the original scale of data, thus the results are easily interpreted [26,28]. Therefore, GLM with a log link function and gamma error distribution were used to analyze the association of costs with DR in this study.

All statistical analysis was performed using STATA version 14.0 (STATA Corporation, College Station, Texas, USA). A two-tailed p value of less than 0.05 was considered as statistically significant.

## Results

### Patient characteristics

In this study, the presence of DR was found in 172 of the 435 (39.5%) patients, including 131 NPDR (51 mild, 62 moderate and 18 severe NPDR) and 41 PDR. **Table 1**summarizes the clinical and biochemical characteristics of T2DM patients stratified by the presence of DR. DR patients were older, have longer duration of diabetes, and higher HbA1c, compared with no DR patients. The percentage of current and former smokers, neuropathy, CKD and commonly used medications in diabetes (e.g., insulin or/and oral glycemic medications, RAS medications) were significantly higher in DR than no DR patients (**Table 1**).

**Table 1:** Clinical and biochemical characteristics of individuals with T2DM stratified by presence of DR (n = 435).

Variables	DR (172)	Non-DR (263)	All (435)	P-value
**Entry age (yrs)**	55.3 ± 9.3	50.8 ± 12.8	52.6 ± 11.8	<0.001
**Age group**, No., (%)				
21–29	0 (0)	21 (8.0)	21 (4.8)	
30–39	12 (7.0)	30 (11.4)	42 (9.7)	
40–49	25 (14.5)	51 (20.5)	79 (18.2)	
50–59	85 (49.4)	85 (32.2)	170 (39.1)	
60–69	39 (22.7)	64 (24.3)	103(23.7)	
>70	11 (6.4)	9 (3.4)	20 (4.6)	<0.001
**Male gender**, No., (%)	58.1	60.5	59.5	0.232
**Ethnicity**, No., (%)				
Chinese	76 (45.2)	154 (60.9)	230 (54.6)	
Malays	48 (28.6)	36 (14.2)	84 (20.0)	
Indians	44 (26.2)	63 (24.9)	107 (25.4)	0.001
**BMI (kg/m**^**2**^**)**	28.3 ± 5.8	28.3 ± 5.7	28.3 ± 5.7	0.901
**Smoking (current and former)**, No., (%)	29 (26.9)	21 (8.0)	50 (11.5)	0.005
**T2DM burden**				
Duration (yrs)	14.5±9.2	9.3±8.3	11.3±9.0	<0.001
HbA1c (%)	8.4±1.4	7.9±1.5	8.1±1.4	0.009
Oral glycemic medication,^a^ No., (%)	157 (91.8)	240 (91.6)	397 (91.7)	0.938
Insulin, No., (%)	89 (52.5)	83 (31.7)	172 (39.7)	<0.001
RAS medication,^b^ No., (%)	129 (75.0)	150 (57.5)	279 (64.4)	<0.001
**History of CVD**				
IHD,^c^ No., (%)	23 (13.8)	30 (11.4)	53 (12.5)	0.602
Stroke No., (%)	15 (8.8)	8 (3.0)	23 (5.6)	0.057
**CKD,**^d^ No., (%)	135 (78.5)	103 (39.2)	238 (54.7)	<0.001
**Neuropathy,**^e^ No., (%)	46 (27.5)	10 (4.0)	56 (13.5)	<0.001

^a^Usage of insulin secretagogues, Rosi-/Pio-glitazone or metformin

^b^Renin-angiotensin system (RAS) medication, angiotensin-converting-enzyme or angiotensin receptor blockers

^c^Ischemic heart disease (IHD), blockade of arteries to the heart, heart Attack, balloon angioplasty of blocked artery of the heart, or heart bypass operation

^d^CKD, eGFR <60 ml/min/1.73m^2^ AND/OR ACR≥30mg/g

^e^Neurothesiometer reading>25 V or monofilament sensory test result below 8 out of 10 points on either side of the feet.

HbA1C, hemoglobin A1c; BMI, body mass index; HDL-C, high density lipoprotein cholesterol; CVD, cardiovascular disease; CKD, chronic kidney disease

### Cost difference by the presence of DR

**Fig 1:**
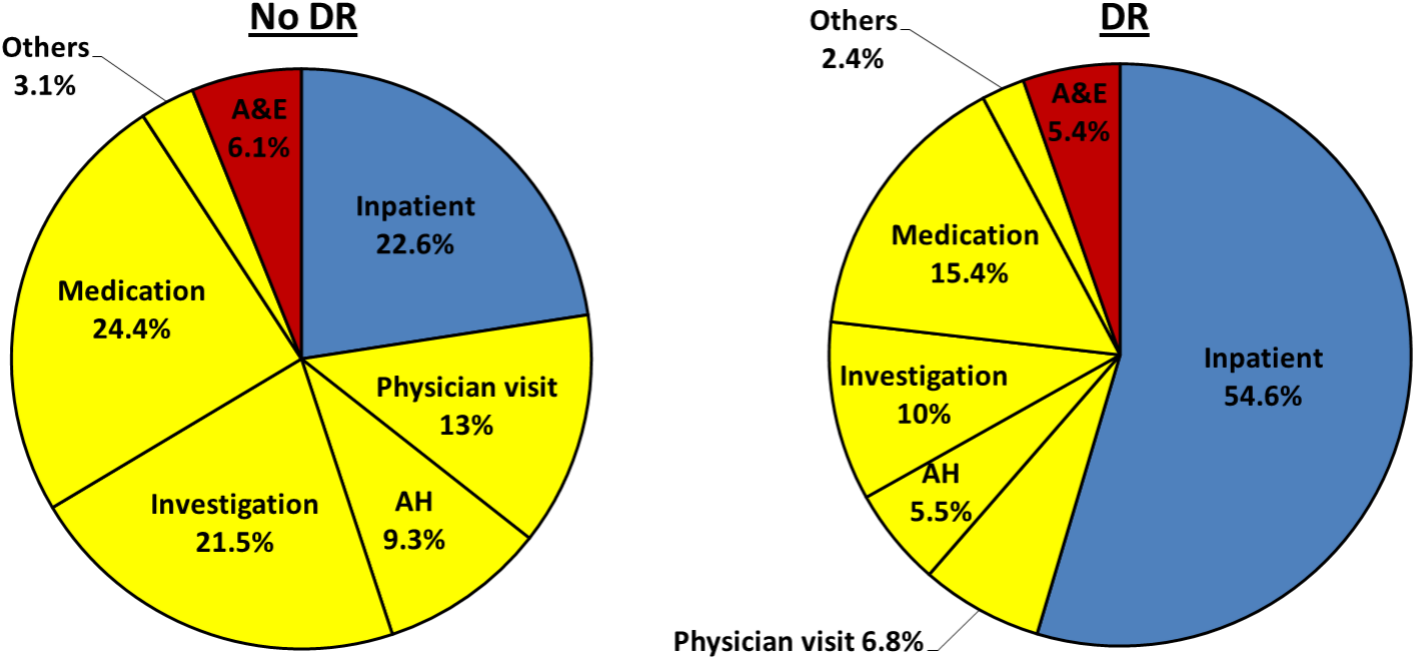
Distribution of cost. (A) cost in DR (B) cost in non-DR patients.

The median of total cost in DR [median (IQR), SGD2012.0 (1111.2–4192.3)] was significantly higher than that in patients without DR [SGD1158.1 (724.1–1838.9)] (p<0.001). The median of costs for inpatient, outpatient and A&E service were all significantly higher in DR than those in non-DR patients (**Table 2**). The corresponding cost ratios for the inpatient, outpatient and A&E relative to no DR patient were 4.4, 1.5 and 2.7, respectively. Of the total costs in DR patients, the main drivers were inpatient (49.8%) and outpatient costs (42.0%), while A&E service cost constituted a small proportion (8.2%). The largest driver of total cost for non-DR patients was outpatient costs (67.6%), while inpatient costs (25.6%) and A&E services (6.8%) accounted for a small portion (**Fig 1**).

**Table 2:** The total cost and cost distribution of individuals with T2DM stratified by presence of DR (n = 435).

	DR (172)		Non-DR (263)				
Costs (SGD)	Mean (SD)	Median (IQR)	Mean (SD)	Median (IQR)	Cost difference	Cost ratio	P-value
**Total medical cost**	4240.8	2012.0	1827.6	1158.1	2413.2	2.3	**<0.001**
	(5868.9)	(1111.2–4192.3)	(3938.8)	(724.1–1838.9)			
**Inpatient cost**	2321.2	0	528.4	0	1792.8	4.4	**<0.001**
	(5064.6)	(0–2056.6)	(3561.35)	(0–0)			
**Outpatient cost**	1705.9	1383.9	1135.8	1022.3	570.1	1.5	**<0.001**
	(1331.8)	(884.6–2085.5)	(791.9)	(564.4–1427.3)			
**A&E cost**	381.3	0	141.2	0	240.1	2.7	**0.006**
	(1123.7)	(0–389.2)	(396.1)	(0–101.4)			

A&E, accident and emergency

### Cost differences by the severity of DR

**Fig 2:**
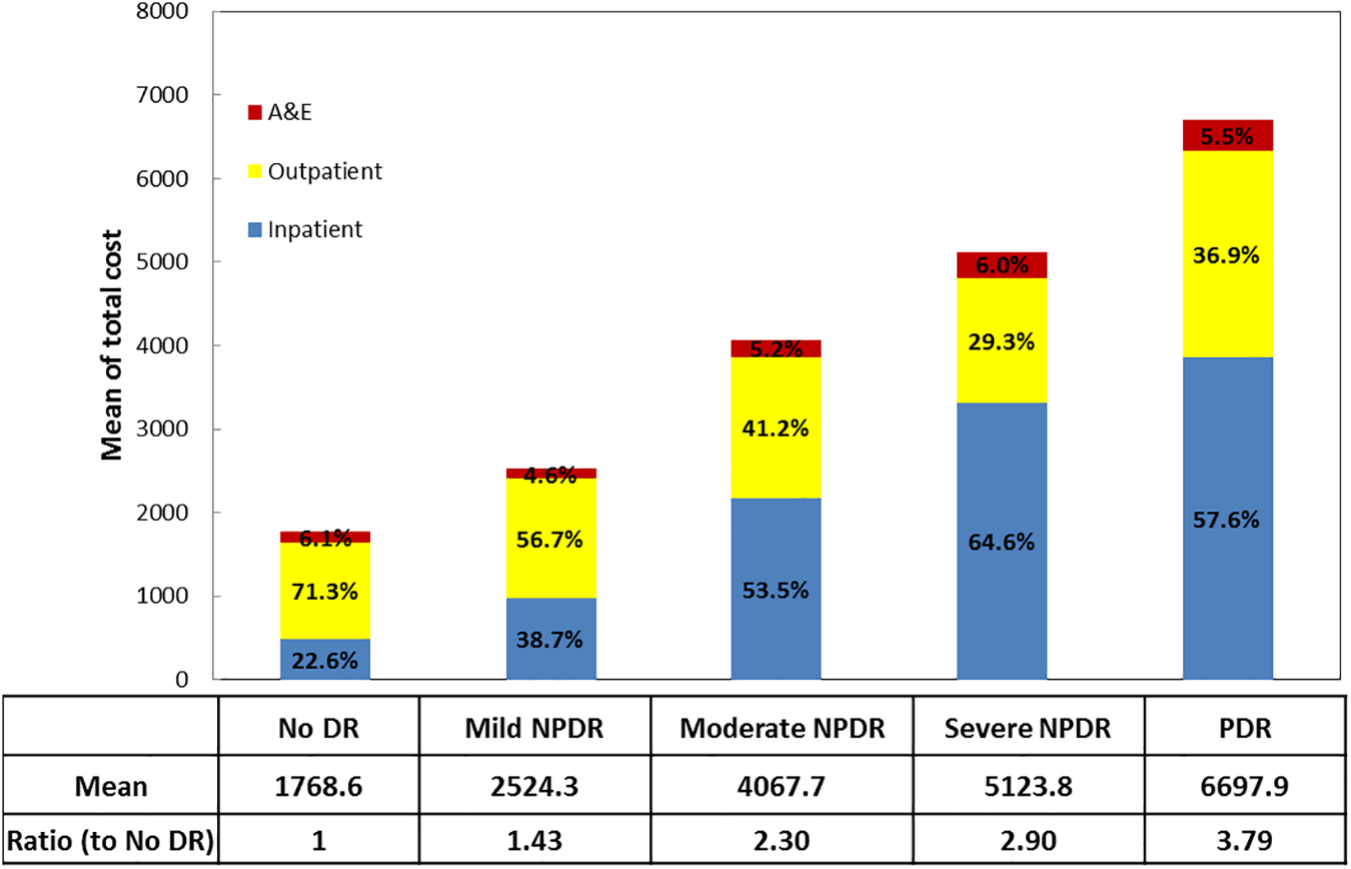
The total cost and distribution categorized by the severity of DR.

When DR patients were further categorized by the severity of DR, the corresponding median total costs (SGD) for mild, moderate, and severe NPDR, and PDR were [1167.1 (895.4–2012.0)], [2212.0 (1215.5–3825.5)], [2717.5 (1444.0–6310.7)], and [3594.8.1 (1978.4–8427.7)], respectively (P<0.001). The corresponding cost ratio for mild, moderate and severe NPDR, and PDR relative to no DR were 1.4, 1.9, 2.8 and 3.8, respectively (**Fig 2**).

### Factors affecting total cost

**Table 3**shows the factors associated with the total cost using generalized linear models. After adjustment, DR severity was significantly associated with the total cost. The corresponding ratio of total cost relative to non-DR patients was 1.1 (p = 0.827 for mild NPDR), 1.8 (p = 0.003 for moderate NPDR), 2.0 (p = 0.031 for severe NPDR) and 2.3 (p<0.001 for PDR), respectively. The other factors affecting the total cost include smoking (ratio = 1.7, p = 0.019), neuropathy (ratio = 1.9, p = 0.001) and CKD (ratio = 1.4, p = 0.019).

**Table 3:** Association of medical costs and the severity of DR (n = 435).

Variables	β (95% CI)	P value	Ratio to Ref.
**Entry age (yrs)**	0.004 (-0.008–0.016)	0.531	
**Male gender**	0.111 (-0.163–0.386)	0.426	
**Ethnicity**			
Chinese	Ref.		
Malay	0.057 (-0.297–0.412)	0.751	
Indian	0.027 (-0.280–0.335)	0.863	
**HbA1c (%)**	-0.008 (-0.100–0.084)	0.870	
**T2DM duration (yrs)**	-0.012 (-0.030–0.005)	0.170	
**Smoking (current and former)**	0.511 (0.0824–0.941)	**0.019**	1.7
**Neuropathy**	0.663 (0.265–1.062)	**0.001**	1.9
**CKD**	0.355 (0.058–0.653)	**0.019**	1.4
**Insulin**	0.271 (-0.016–0.559)	0.065	1.3
**RAS medication**	0.098 (-0.173–0.370)	0.477	
**DR severity**			
No DR	Ref.		
Mild NPDR	0.041 (-0.324–0.406)	0.827	1.1
Moderate NPDR	0.585 (0.203–0.968)	**0.003**	1.8
Severe NPDR	0.708 (0.064–1.353)	**0.031**	2.0
PDR	0.854 (0.384–1.323)	**<0.001**	2.3

HbA1C, hemoglobin A1c; T2DM, type 2 diabetes; CKD, chronic kidney disease; RAS: renin-angiotensin system; NPDR, non-proliferative diabetic retinopathy; PDR, proliferative diabetic retinopathy; PDR

## Discussion

In the cost-of-illness study among the multi-ethnic T2DM Asian cohort, we found that the presence and severity of DR was associated with increased direct medical costs. To the best of our knowledge, this is the first study to examine the relationship between DR (prevalence and severity) and direct medical cost incurred in Singapore. Our results suggest that preventing progression of DR may reduce the economic burden of DR.

We observed significantly increased burden of other co-morbidities with DR severity, such as CKD (39.2% vs. 74.8% vs. 90.2% for no DR vs. NDR vs. NPDR, p<0.001) and neuropathy (4.0% vs. 23.4% vs. 41.0% for no DR vs. NDR vs. NPDR, p<0.001), suggesting DR is a proxy for total disease burden in T2DM patients. The inpatient cost occurred in 34.4% of NPDR and 68.3% of PDR patients, and it becomes a main driver of the total cost for DR patients, which might be attributable to the hospitalization for other co-morbidities. The high cost of inpatients service has been reported to be strongly correlated with length of stay (LOS), suggesting that attempts to reduce LOS through expediting services or reducing unnecessary utilization of diagnostic tests, may reduce the overall medical cost [31,32]. In outpatient costs, the highest relative ratio to no DR patients is 1.2 and 1.5 for medications and other services, individually. These may include introductions of more prescription drugs (e.g. avastin), visual or mobility aids, and other relevant devices for DR patients.

The mean annual direct medical cost (SGD2866.7) in 2014 in our study is generally in line with the previously reported cost (SGD2034.6) in 2010 in Singapore [17]. However, in that study, the presence of DR is not significantly associated with cost [17]. One possible reason is that the majority of cost data were obtained from polyclinic patients, whose conditions were less complex compared with those for patients visiting KTPH, a regional hospital in our study. This may explain their better clinical profile (26.1±4.7 vs. 28.3±5.7kg/m^2^ for BMI; 7.3±1.2 vs. 8.1±1.4% for HbA1C) and lower DR prevalence (15% vs. 39.4%) compared to our study. The Singapore epidemiology of eye diseases study found that five in six people who have DR or damage to the blood vessels in the eye are unaware of the condition [10]. Therefore, the lower DR prevalence in that study may also be caused by the unawareness and under-reporting. The low statistical power due to small sample size of DR patients (n = 75) may have limited the analysis on the influence of DR on cost as well.

The direct medical cost per patient in this study appears to be lower than cost among T2DM patients in Western [11–14] and Asian countries [16]. However, the observed association of cost with DR, and cost ratio for DR relative to non-DR is consistent with those studies. In Switzerland, the mean direct medical cost per T2DM patient in 1998 (CHF3,508, US$2543.9) is slightly higher than ours (US$2219.4). However, the cost ratio of DR to non-DR in our study (ratio = 2.32) agreed very well with that study (ratio = 2.26) [11]. In a USA study using claim data from 1999 to 2003 among employees, the mean direct medical cost in DR (US$14,670) is significantly higher than those without DR (US$9,524) after adjustment for demographic characteristics and comorbidities [14]. Within the DR subgroups, PDR employees cost over twice as much on average as NPDR, which agrees with ours (ratio = 1.85) [14]. In Germany, the ratio of corresponding cost attributable to DR for mild, moderate, severe NPDR, and PDR relative to no DR were 1.2, 2.5,3.2 and 7.4, respectively, which is also consistent with the increasing trend observed in our study [12]. The only longitudinal study in Taiwan found that PDR patients had the highest baseline costs (US$3,632) than NPDR (US$2331.3) or non-DR patients (UD$1921.1) in 2000 [16]. After 4-year follow up, patients who progressed from NPDR to PDR experienced the greatest increase in costs at US $3482 compared with those remained stable or progressed from non-DR to any DR [16]. Considering the difference in DR classification and treatment, cost estimation methodology, reimbursement systems, and inflation, comparing our results on the healthcare costs with results in other countries is difficult. However, all these cost-of-illness studies demonstrated that DR carried a significant economic burden for the diabetic patient, and the cost increased substantially with the presence and severity of DR.

The advantage of this study includes accurate DR status obtained by fundus imaging and inclusion of demographics and clinical characteristics. Although secondary data (i.e., register- or claim-based dataset) allows for large sample size, it includes a risk of misclassification or low accuracy, and incomplete patient information [33]. Such problems are not an issue in the present study. When comparing DR status obtained by self-report and by fundus photograph imaging, we found the overall accuracy of self-report DR status is 73.2%, which is consistent with the accuracy of claims data in Taiwan study (74.6%) [34]. Among the 300 patients self-reporting non-DR, 80 (26.7%) were classified as NPDR (n = 71) or PDR (n = 9) by photographs grading, which indicates clear under-reporting of DR, especially for asymptomatic NPDR, using data from electronics patient records, case-notes or questionnaire rather than fundus imaging upon study enrolment. Previous studies relying on claim data were unable to retrieve information, such as demographics (e.g., ethnicity) and T2DM-related variables (e.g., duration of diabetes, Hba1C). In our study, we compared the cost by the presence of severity of DR after taking into account the effect of these confounding factors. Another advantage of this study is that the cost was retrieved from hospital administrative data, instead of multiplying each resource by unit costs, which may reflect the actual expenses incurred more accurately.

Several limitations have also been identified. First, the sample size is relatively small compared with studies using secondary dataset. Second, the total cost for T2DM is composed of three components, including direct medical and non-medical (e.g., transportation, relocation and informal care) costs, indirect costs (e.g., productivity losses) and intangible costs (e.g., psychological pain, discomfort and distress related to diabetes) [35]. American Diabetes Association estimated that indirect costs account for 33% of total costs for the general diabetes population [36]. Patients with microvascular complications, such as DR, may even have a higher proportion of indirect [14] and intangible costs [37]. In our study, we only estimated direct medical cost using hospital administrative data, which does not contain information for non-medical or indirect costs. More studies are needed to understand the contribution of other cost components. Finally, our study was based on diabetes patients in a secondary hospital, whose conditions may be different from diabetes patients at national level. For example, Singapore National Health Survey in 2010 reports better profiles than ours, such as lower fasting plasma glucose level (7.6 vs. 8.9mmol/l) and less poor glycemic control (defined as HbA1C≥8.0%) (32.2% vs. 49.9%) [38]. Therefore, whether our findings can be extrapolated to a national level remains to be determined.

In conclusion, we found that the presence and severity of DR was associated with increased direct medical costs in a multi-ethnic Asian T2DM cohort in Singapore, suggesting that strategies aimed at preventing progression of DR may reduce the economic burden of DR, as well as other co-morbidities. This information would be useful for further cost-effectiveness studies (i.e., novel DR examination and treatments), and help maximize value for patients and the healthcare system.

## Supporting information

S1 Table Characteristics of selected and unselected individuals with T2DM. (DOCX)

S2 Table Characteristics of individuals with T2DM for analysis and not for analysis. (DOCX)
