# Supplementary material for: Direct medical cost associated with diabetic retinopathy severity in type 2 diabetes in Singapore
Source: PLoS One. 2017 Jul 10;12(7):e0180949. doi: 10.1371/journal.pone.0180949 (PMC5507311; doi:10.1371/journal.pone.0180949)
Supplement: S1 Table — (DOCX) [file pone.0180949.s001.docx]

**S1 Table.** Characteristics of selected and unselected individuals with T2DM

| **Variables** | **Selected (482)** | **Unselected (1,575)** | **P-value** |
| --- | --- | --- | --- |
| **Entry age (yrs)** | 53.1±11.6 | 58.7±10.2 | <0.001 |
| **Male gender (%)** | 58.3% | 48.7% | <0.001 |
| **Ethnicity (%)** |  |  |  |
| Chinese | 52.7% | 50.7% |  |
| Malays | 19.8% | 23.3% |  |
| Indians | 24.6% | 22.6% | 0.362 |
| **Duration of T2DM (yrs)** | 11.6±8.8 | 11.5±8.9 | 0.833 |
| **HbA1c (%)** | 8.1±1.5 | 7.7±2.0 | 0.001 |
| **SBP (mmHg)** | 141.2±18.8 | 141.1±19.4 | 0.876 |
| **DBP (mmHg)** | 82.8±14.1 | 82.1±18.1 | 0.468 |
| **BMI (kg/m^2^)** | 28.2±5.6 | 27.6±5.1 | 0.033 |
| **eGFR (ml/min/1.73 m^2^)** | 90.3±35.5 | 82.9±32.3 | <0.001 |
| **Neuropathy** (%) | 15.0% | 17.3% | 0.281 |
| **IHD (%)** | 11.6% | 12.9% | 0.569 |
| **Stroke (%)** | 5.5% | 3.2% | 0.112 |
| **Current and ex-smokers (%)** | 21.0% | 14.8% | 0.002 |

T2DM, type 2 diabetes, HbA1c, hemoglobin A1c; SBP, systolic blood pressure; DBP, diastolic blood pressure; BMI, body mass index; eGFR: estimated glomerular filtration rate; IHD, ischemic heart disease

p<0.05 was considered as statistically significant
