# Supplementary material for: Direct medical cost associated with diabetic retinopathy severity in type 2 diabetes in Singapore
Source: PLoS One. 2017 Jul 10;12(7):e0180949. doi: 10.1371/journal.pone.0180949 (PMC5507311; doi:10.1371/journal.pone.0180949)
Supplement: S2 Table — (DOCX) [file pone.0180949.s002.docx]

**S2 Table.** Characteristics of individuals with T2DM for analysis and not for analysis

| **Variables** | **Analysis (435)** | **Not for analysis (47)** | **P-value** |
| --- | --- | --- | --- |
| **Entry age (yrs)** | 52.6 ± 11.8 | 57.5±1.4 | 0.007 |
| **Male gender (%)** | 59.5 | 47.8 | 0.112 |
| **Ethnicity (%)** |  |  |  |
| Chinese | 230 (54.6) | 47.8 (22) |  |
| Malays | 84 (20.0) | 10 (21.7) |  |
| Indians | 107 (25.4) | 12 (26.1) | 0.692 |
| **Duration of T2DM (yrs)** | 11.3±9.0 | 10.7±9.1 | 0.503 |
| **Hba1c (%)** | 8.1±1.4 | 7.6±1.1 | 0.018 |
| **BMI (kg/m^2^)** | 28.3±5.7 | 26.6±4.3 | 0.040 |
| **Current and former smokers (%)** | 95 (21.8) | 7 (15.2) | 0.296 |

T2DM, type 2 diabetes, HbA1c, hemoglobin A1c; BMI, body mass index

p<0.05 was considered as statistically significant
